# Trends in Stage and Age at Diagnosis of Oral Squamous Cell Carcinoma Over Time: A Systematic Review and Meta‐Analysis

**DOI:** 10.1111/odi.70403

**Published:** 2026-07-05

**Authors:** Francesco Scilla, Rita Antonelli, Cosimo Rupe, Gioele Gioco, Romeo Patini, Laura Ferrero, Giulio Tedeschi, Paolo Vescovi, Ilaria Giovannacci, Marco Meleti, Carlo Lajolo

**Affiliations:** ^1^ Head and Neck Department, Fondazione Policlinico Universitario A. Gemelli—IRCCS, School of Dentistry Università Cattolica del Sacro Cuore Rome Italy; ^2^ Department of Medicine and Surgery, University Center of Dentistry University of Parma Parma Italy

**Keywords:** age, epidemiology, meta‐analysis, oral squamous cell carcinoma, stage, systematic review, temporal trends

## Abstract

**Objective:**

To evaluate whether the age at diagnosis of oral squamous cell carcinoma (OSCC) has changed over time and to assess whether stage at presentation has varied across decades.

**Methods:**

A systematic review was conducted according to PRISMA guidelines (PROSPERO registration: CRD420251048070). MEDLINE, Scopus, and Web of Science were searched. Studies reporting age at diagnosis and/or stage distribution of OSCC were included.

**Results:**

Twenty‐nine studies (15,911 subjects) were included. A primary meta‐analysis of 11 eligible studies (*n* = 3527) yielded a pooled mean age at diagnosis of 57.42 years (95% CI: 54.51–60.33). A sensitivity analysis (8 studies with sample size ≥ 100 patients, total = 3322) produced a pooled estimate of 55.61 years (95% CI: 52.32–58.91), suggesting a potentially meaningful shift toward younger age at diagnosis in larger cohorts. Early‐stage disease (I–II) accounted for 54.2% of cases and advanced‐stage disease (III–IV) for 45.8%. Variability in staging systems (AJCC/UICC editions) and reporting formats limited direct cross‐decade comparisons.

**Conclusions:**

Current evidence is compatible with a modest, population‐dependent trend toward younger age at diagnosis in selected contemporary cohorts. Standardized staging, harmonized reporting, and age‐stratified risk‐factor analyses are needed to clarify whether emerging young‐onset OSCC reflects changing exposure profiles, distinct tumor biology, or both.

**Trial Registration:**

PROSPERO Registration: CRD420251048070

## 1 Introduction

Oral Squamous Cell Carcinoma (OSCC) is the most frequent malignant neoplasm of the oral cavity, involving cancers of the lip, floor of the mouth, oral tongue, buccal mucosa, upper and lower gingivae, hard palate, and retromolar trigone, with the oral tongue representing the most affected subsite (Gamez et al. 2018). In 2022, an estimated 389,485 new cases were diagnosed worldwide (2% of all sites) and 188,230 related deaths, showing increases of 9.7% and 6.1%, respectively, compared to 2018 (Santos et al. 2016; Bray et al. 2018, 2024). Furthermore, despite advances in therapeutic options, the overall 5‐year survival rate remains below 50% (Antonelli et al. 2024). Traditional theories suggested that OSCC usually affects individuals in their 50s and 60s, especially men with a history of long‐term exposure to traditional risk factors such as tobacco and alcohol use (Xu et al. 2019). Approximately 95% of OSCC occurs after 45 years (Gamez et al. 2018).

However, several epidemiological studies have reported a possible shift in the age of onset, with a rising incidence of cases among young adults (arbitrarily aged less than 45 years). A Dutch cancer registry analysis covering the period from 1989 to 2018 reported a significant annual increase of 2.4% in OSCC incidence among patients aged 20–34 years, contrasting with a decrease in the 35–44 age group. In patients over 60 years, incidence rates also increased overall, predominantly in smokers and alcohol consumers (Al‐Jamaei et al. 2022). Similarly, data from Korea show a notable increase in oral cancer incidence between 2001 and 2021 among female patients aged 45 or younger, with particularly pronounced rises in tongue cancer (Shin et al. 2025). Moreover, analysis of U.S. Surveillance, Epidemiology, and End Results (SEER) database for the years 1975 to 2007 revealed an alarming upward trend in oral tongue SCC among young women (aged 18–44), with an annual percentage change (APC) of +4.0% (Patel et al. 2011). The reasons underlying this possible epidemiological shift remain not completely understood. Classical risk factors do not fully explain the phenomenon, since many younger patients report minimal or no history of tobacco or alcohol use (Llewellyn et al. 2001). Relevant molecular characteristics of OSCC in young adults have been identified, suggesting potential differences in tumor biology. However, data remain limited and further research is needed to explore additional factors, such as prior viral infections (e.g., Human Papilloma Virus and Epstein–Barr Virus), genetic predisposition, immunodeficiency, oral microbiome, and yet‐unrecognized environmental carcinogens (Llewellyn et al. 2001; Lenoci et al. 2024). Clinicopathological and prognostic features in younger OSCC patients also warrant careful consideration. A recent meta‐analysis comparing younger versus older OSCC patients (studies from 2014 to 2019) confirmed that younger individuals tend to have lower exposure to tobacco but present with comparable tumor differentiation and stage. Notably, the younger cohort displayed a higher recurrence rate (42%), despite similar overall survival (Mohideen et al. 2021).

Accordingly, this systematic review aimed to determine whether the age at diagnosis of OSCC has changed over time, specifically whether more recent cohorts show a shift toward younger patients, and to examine whether stage at diagnosis has varied across decades. Such findings could have important implications for public health strategies, early detection, and therapeutic approaches for this emerging patient population. Moreover, they may shed light on the presence of risk factors that are currently unrecognized or insufficiently investigated, thereby guiding future etiological research.

## 2 Materials and Methods

The present systematic review followed the Preferred Reporting Items for Systematic Reviews and Meta‐Analyses (PRISMA) statement. The review protocol was registered in the PROSPERO database (CRD420251048070).

### 2.1 PECOS

The following question was developed according to the Population, Exposure, Comparison, Outcome, and Study design (PECOS).

- Population: patients with a histopathological diagnosis of oral squamous cell carcinoma.
- Exposure: Calendar period of OSCC diagnosis (year‐ or decade‐based strata, as reported by each study).
- Comparison: the age at diagnosis and the clinical stage at diagnosis reported in earlier time periods were compared with those reported in more recent periods to assess temporal changes.
- Outcome: the primary outcomes were to evaluate whether the age at OSCC diagnosis has changed over time and whether the stage at presentation has changed, with particular interest in the potential increase of OSCC cases among younger individuals.
- Study design: retrospective or prospective cohorts, cross‐sectional epidemiological studies, and cancer registry analyses reporting diagnostic age and/or staging at the time of OSCC diagnosis were included.

### 2.2 Focused Question

The question, formulated according to the PECOS criteria, that this review attempts to answer is: “Have age at diagnosis and stage at presentation of oral squamous cell carcinoma changed over time, and do current trends suggest an increasing occurrence of the disease in younger patients?”

### 2.3 Inclusion Criteria

The search was limited to studies published in English, Italian, French, and Spanish without any restriction regarding the year of publication. Studies were considered eligible if they reported OSCC age at diagnosis and/or stage at diagnosis in relation to a defined diagnostic time frame or calendar period. Temporal comparisons were subsequently interpreted according to the way each included study reported its diagnostic periods. No predefined calendar cut‐offs were imposed to define “earlier” versus “more recent” periods; temporal comparisons were interpreted according to the stratification reported within each included study and subsequently grouped by decade of publication for subgroup synthesis. For studies reporting data from broader head and neck cancer populations, only data pertaining specifically to oral cavity squamous cell carcinoma were extracted.

### 2.4 Exclusion Criteria

The following exclusion criteria were applied: studies with a population of fewer than 10 patients, in vivo or in vitro animal model studies, systematic reviews, letters to editor, conference proceedings, personal communications and articles published in languages other than English, Italian, French, and Spanish and studies reporting OSCC cases over a long, aggregated diagnostic period (e.g., multi‐decade series) without providing diagnosis‐year information at the individual level or stratified summaries by calendar period (e.g., decade or predefined time intervals), thus preventing temporal trend analyses. Studies were excluded if they reported OSCC cases over long aggregated diagnostic periods without providing stratified data by calendar period or otherwise usable temporal comparisons.

No universal calendar cut‐offs were imposed a priori. “Earlier” and “more recent” periods were defined according to the temporal stratification reported in each included study. For subgroup synthesis, studies were subsequently grouped by decade of patient recruitment/diagnosis.

### 2.5 Search Strategy

The electronic searches were last performed on 04 May 2026 in three databases (PubMed, Scopus, and Web of Science). The search strategy was initially developed for PubMed using a combination of keywords and MeSH terms and was subsequently adapted for the other databases: ((((adult[MeSH Terms] OR aged[MeSH Terms] OR patient[MeSH Terms]) AND (mouth neoplasm[MeSH Terms] OR oral cancer[MeSH Terms] OR cancer, squamous cell[MeSH Terms] OR oral neoplasm[MeSH Terms])) AND (young adult[MeSH Terms] OR middle aged[MeSH Terms])) AND (cancer early diagnosis[MeSH Terms] OR cancer staging[MeSH Terms] OR neoplasm staging[MeSH Terms] OR early diagnosis[MeSH Terms] OR neoplasm grading[MeSH Terms])) AND (mouth[MeSH Terms]). The complete search strategies for all databases, together with the number of retrieved records, are reported in Table S1.

Duplicate records were identified and removed using Zotero reference management software (Zotero 2026).

### 2.6 Manual Search

A manual search was performed on 05 May 2026 by screening the tables of contents of the following journals: Oral Oncology, Oral Diseases, Lancet Oncology, and Journal of Hematology and Oncology. Articles were screened using the following terms: oral squamous cell carcinoma, oral cancer, age at diagnosis, stage at diagnosis. Potentially relevant articles addressing OSCC, age at diagnosis, stage at diagnosis, and temporal trends were assessed for eligibility.

### 2.7 Search of Unpublished Articles

Unpublished literature was searched in the U.S. National Institutes of Health clinical trials registry and the European Multidisciplinary Database to identify incumbent studies and gray literature. In addition, bibliographic references of all included articles and reviews were similarly checked to identify additional potentially relevant studies and increase the sensitivity of the search.

### 2.8 Study Selection

Based on the inclusion criteria, two authors independently and in duplicate (F.S. and R.A.) screened the titles and abstracts of the articles found. Full‐text versions were retrieved for articles that appeared to meet the inclusion criteria or for those reporting insufficient data to make a clear decision. The two authors then independently assessed the full texts to determine whether the articles met the above‐mentioned criteria. Disagreements were resolved through discussion between the two authors, and when consensus could not be reached, a third senior author (R.P.) was consulted. Agreement between reviewers was calculates using Cohen's kappa coefficient, with *k* > 0.75 considered excellent, *k* = 0.40–0.74 considered fair to good, and poor when it was less than 0.4 (Landis and Koch 1977). All included papers underwent to data extraction and quality assessments, while irrelevant articles were excluded, and the reasons for exclusion were as described. Articles which were excluded after full‐text evaluation along with reason for exclusion, are reported in Table S2.

### 2.9 Extraction Data

The data were collected using a purpose‐built data extraction form. In cases where the publication did not provide all the necessary data, the corresponding author was contacted by e‐mail to obtain the missing data. In case of authors' disagreement about information to be drawn from publications, a discussion was opened, which, in cases of further disagreement, required the intervention of the third author. In cases of redundant publications, the most recent article and the one with the largest follow‐up were included. When means and standard deviations were not directly reported, they were estimated from medians and ranges or interquartile ranges using the methods described by Wan et al. (2014) and Luo et al. (2018). For each included study, the AJCC/UICC edition used for staging was recorded as a covariate. Where a study reported classifications under more than one edition for the same cohort, data from both editions were extracted separately to enable direct quantification of stage migration.

### 2.10 Quality Assessment

The risk of bias (RoB) in the included studies was assessed independently and in duplicate by two authors as part of the data extraction process. An assessment of RoB was undertaken using the Newcastle–Ottawa Scale (NOS), which assesses three domains (selection, comparability, and outcome) through 8 items (Wells et al. 2000). The presence of each parameter was recorded with a green mark, while absence was recorded with a red mark. Papers with 1–3 green marks were classified as high risk of bias, those with 4–6 green marks were classified as medium risk, and those with 7–8 green marks were classified as low risk. A supplemental analysis was performed independently by the two examiners regarding the overall quality of the evidence for any performed meta‐analysis using the classification of the Oxford Center for Evidence‐Based Medicine (OCEBM) levels for diagnosis (2011) (Oxford Centre for Evidence‐Based Medicine 2009).

Any disagreement between the two reviewers (F.S. and R.A.) was solved by discussion with the author supervisor (R.P.). In case of at least 10 articles included publication bias was assessed by visual inspection of funnel plots generated using MetaAnalysisOnline.com (Fekete and Győrffy 2025).

### 2.11 Heterogeneity Assessment

The OpenMeta software was used for assessing the heterogeneity of the studies included in any conducted meta‐analysis (OpenMeta Inc. Zug, Zug, Switzerland). Statistical heterogeneity was assessed using Cochran's *Q* test and quantified using the *I* ^2^ statistic. A *p* value < 0.10 for the *Q* test was considered suggestive of statistically significant heterogeneity. The *I* ^2^ statistic was interpreted according to Cochrane guidance as follows: The magnitude of heterogeneity was interpreted according to the following thresholds: 0%–40% negligible, > 40%–60% moderate, > 60%–75% substantial, and > 75% considerable, as suggested by Higgins et al. (Higgins and Thompson 2002). These thresholds were interpreted cautiously in conjunction with clinical and methodological heterogeneity.

### 2.12 Data Analysis

Descriptive characteristics of the studies are expressed as means/medians and/or frequencies, as appropriate, depending on the variables. Meta‐analyses were performed only when there were studies comparing similar groups and reporting the same outcomes. Given the expected clinical and methodological heterogeneity across studies, random‐effects models were used as the primary analytical approach. Fixed‐effect models were additionally explored as sensitivity analyses. A forest plot was created to illustrate the effects on the meta‐analysis of individual studies and the overall estimate. OpenMeta‐analyst was used to perform all analyses. The cut‐off value of significance was set at *p* < 0.05.

A subgroup meta‐analysis was performed stratifying studies by decade of publication: studies covering excessively broad or overlapping diagnostic periods were systematically excluded from the quantitative synthesis. As a result, in the included studies the publication decade closely approximates the decade of patient diagnosis, minimizing the potential discrepancy between these two stratification approaches. A sensitivity analysis was conducted restricting the quantitative synthesis to studies with a sample size of at least 100 patients in order to assess the robustness of the pooled estimate and reduce the potential influence of small, single‐centre series on the overall result. Studies with *n* < 100 were retained in the primary meta‐analysis but excluded from this secondary analysis. The pooled estimates from the primary and sensitivity analyses were compared to evaluate consistency of findings.

A subgroup meta‐analysis stratified by geographic region (Americas, Europe, Asia) was additionally performed to explore the potential contribution of geographic heterogeneity to the observed variability in mean age at diagnosis.

To improve comparability of staging data across studies using different AJCC/UICC editions and to minimize the risk of stage migration bias, staging information was harmonized into two broad categories: early‐stage disease (Stage I–II) and advanced‐stage disease (Stage III–IV) wherever complete stage distributions were available. To quantify the potential contribution of staging system heterogeneity to observed differences in stage distributions, studies providing classification of the same patient cohort under both a pre‐8th edition and the AJCC 8th edition framework were identified.

## 3 Results

### 3.1 Study Selection

A flowchart of the search strategy and study selection is shown in Figure 1.

**FIGURE 1:**
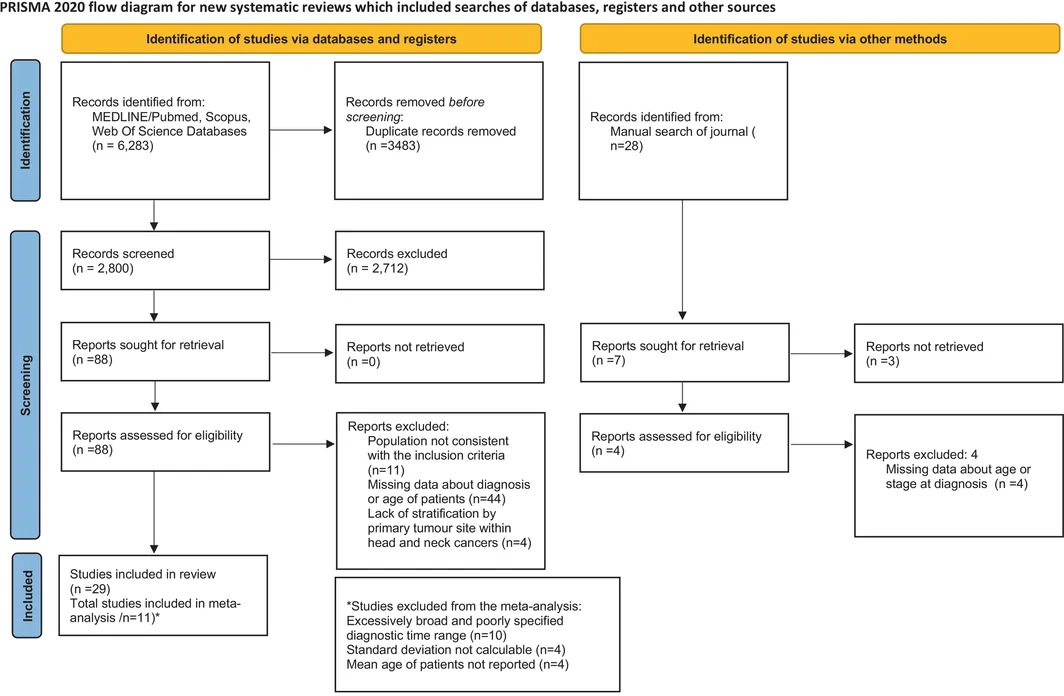
Flowchart of the selection of the studies for the review. *Source:* Page et al. (2021).

A total of 2906 articles was identified, with 2878 found through electronic searches and 28 found through other sources. Out of the 2800 studies that resulted after removal of the duplicates, 2712 were excluded as a result of title and abstract reading (inter‐reader agreement, *k* = 0.79). Eventually, out of the 88 articles that remained to be evaluated in the full‐text, 29, met the inclusion criteria and were included in the qualitative analysis (Gamez et al. 2018; Jawad et al. 2020; Yung et al. 2021; Yu et al. 2023; Jonasson et al. 2023; Morelatto et al. 2007; Anneroth et al. 1986; Toledo et al. 2021; Baba et al. 2021; Lubek et al. 2013; Boland et al. 2013; Dipti et al. 2023; Tagliabue et al. 2022; Urist et al. 1987; Suzuki et al. 2007; Gadbail et al. 2017; Fries et al. 1980; Jehn et al. 2019; Singhavi et al. 2020; Oji and Chukwuneke 2007; Ribeiro et al. 2000; Lin and Bhattacharyya 2009; Kuriakose et al. 2000; Koo et al. 2013; Groome et al. 2011; Alonso et al. 2018; Siegelmann‐Danieli et al. 1998). Among these, 11 were also included in the quantitative analyses (meta‐analysis) (Yu et al. 2023; Baba et al. 2021; Boland et al. 2013; Dipti et al. 2023; Tagliabue et al. 2022; Gadbail et al. 2017; Singhavi et al. 2020; Ribeiro et al. 2000; Siegelmann‐Danieli et al. 1998; Abrahão et al. 2020, 2018). Eighteen studies were not included in the meta‐analysis for methodological reasons: 10 retrospective studies covered excessively long diagnostic time frames (exceeding 10 years); 4 studies did not report sufficient data to allow calculation of standard deviations; 4 studies did not provide mean values, nor sufficient information to derive them. The corresponding authors of these studies were contacted to request additional information or access to raw data; however, no responses were received; 49 were excluded from both quantitative and qualitative analyses.

### 3.2 Study Characteristics

This systematic review includes 27 retrospective cohort studies (Gamez et al. 2018; Jawad et al. 2020; Yung et al. 2021; Yu et al. 2023; Jonasson et al. 2023; Morelatto et al. 2007; Anneroth et al. 1986; Toledo et al. 2021; Baba et al. 2021; Lubek et al. 2013; Boland et al. 2013; Dipti et al. 2023; Tagliabue et al. 2022; Urist et al. 1987; Suzuki et al. 2007; Gadbail et al. 2017; Fries et al. 1980; Jehn et al. 2019; Singhavi et al. 2020; Oji and Chukwuneke 2007; Ribeiro et al. 2000; Lin and Bhattacharyya 2009; Kuriakose et al. 2000; Koo et al. 2013; Groome et al. 2011; Alonso et al. 2018; Siegelmann‐Danieli et al. 1998), 1 cross‐sectional study (Abrahão et al. 2020) and 1 multicentre prospective study (Abrahão et al. 2018). Among these, focusing on those included in the meta‐analysis: eight were retrospective studies, one was a cross‐sectional study and one was prospective. General information on the included papers (i.e., study design, year of publication, country, number of patients, mean age and SD, gender of patients, range of time of diagnosis, tumor site, staging of the tumor) are reported in Table 1.

**TABLE 1:** Main findings from the included studies.

Title	Authors	Year	Country	Study desing	No. of patients	Mean age; ±SD	Gender (M/F)	Time of diagnosis	Tumor site	Staging	Follow‐up
Treatment outcomes of squamous cell carcinoma of the oral cavity in young adults	Mauricio E. Gamez, et al.	2018	USA	R	124	33.8; ±5.4	74/50	1980–2014	Tongue: 107 Other (n.a.): 17	(AJCC7) Stage I: 65 Stage II: 29 Stage III: 9 Stage IV: 12 Missing data: 9	9 years
Survival rates according to tumor location in patients with surgically treated oral and oropharyngeal SCC	Jehn P. et al.	2019	Germany	R	1486	60.0; ±12.5	72.4/27.6	NR	Mouth floor and mandible: 840 (56.5%) Anterior tongue: 223 (15%) Base of tongue & oropharynx: 135 (9.1%) Palate: 126 (8.5%) Maxilla: 93 (6.3%) Buccal mucosa & vestibule: 69 (4.6%)	(UICC) T1–T2: 893 T3–T4: 541 52 NR N0: 832, N1: 611, 44 NR M0: 1427, M1: 13, 46 NR	≥ 18.0 years 5‐year SR 54.1% Buccal mucosa: 68.2% Anterior tongue: 62.8% Mouth floor & mandible: 57.1% Palate: 55% Maxilla: 42.4% Base of tongue/oropharynx: 38.8%
Comparison of the seventh and eighth editions AJCC classification system in oral cavity squamous cell cancers	Singhavi HR	2020	India	R	863	50.91; ±19.8	701/162	2012–2014	Oral tongue: 365 (42.3%) Buccal mucosa: 277 (32.1%) Retromolar trigone: 76 (8.8%) Lower alveolus: 114 (13.2%) Upper alveolus: 22 (2.5%) Hard palate: 7 (0.8%) Mouth floor: 2 (0.2%)	AJCC7 Stage I: 216 Stage II: 232 Stage III: 134 Stage IV: 281 AJCC8 Stage I: 128 Stage II: 212 Stage III: 191 Stage IV: 332	24 months
Oral cancer in Enugu, Nigeria, 1998–2003	Oji C. and Chukwuneke	2007	Nigeria	R	81	48.4; ±16.1	48/33	1998–2003	Lip: 12 Tongue: 24 Mouth floor: 17 Other: 28	Stage III: 70 (86%) Stage IV: 11 (14%)	NR
A population‐based analysis of verrucous carcinoma of the oral cavity	Alonso J.E., et al.	2018	USA	R	1481	69.5; ±13.1	748/733	1973–2012	Tongue: 428 (28.9%) Alveolar ridge: 317 (21.4%) Buccal mucosa: 281 (19.0%) Lip: 146 (9.9%) Mouth floor: 94 (6.3%) Hard palate: 79 (5.3%) Unspecified oral cavity: 76 (5.1%) Retromolar trigone: 60 (4.1%)	(AJCC7) Stage I: 208 (48.8%), Stage II: 129 (30.3%), Stage III: 50 (11.7%) Stage IV: 39 (9.2%)	NR
Predictors of survival after head and neck squamous cell carcinoma in South America: The InterCHANGE study	Abrahão R., et al.	2020	South America: Argentina, Brazil, Colombia, Uruguay	P	1463	59.8; ±12.2	1185/278	2011–2017	OC: 599 (40.9%), OPC: 408 (27.9%), LC: 378 (25.8%), HC: 78 (5.3%)	(AJCC7) Stage I: 174 (58 LC, 3 HC, 95 OC, 18 OPC) Stage II: 190 (46 LC, 9 HC, 102 OC, 33 OPC) Stage III: 287 (94 LC, 14 HC, 111 OC, 68 OPC) Stage IV: 812 (180 LC, 52 HC, 291 OC, 289 OPC)	1.7 years 3‐year SR: LC 56.0% OC 54.7% OPC 48.0% HC 37.8%
Impact of comorbidity, symptoms, and patients' characteristics on the prognosis of oral carcinomas	de Cassia Braga Ribeiro K, et al.	2000	Brazil	R	110	57.5; ±8.2	93/17	1990–1994	Tongue: 59 Mouth floor: 51	(UICC4, 2nd rev) Stage I: 14 Stage II: 20 Stage III: 30 Stage IV: 46	> 5 years 5 years SR: 33.4%
Tumor volume estimated by computed tomography as a predictive factor in carcinoma of the tongue	Kuriakose M.A., et al.	2000	USA	R	20	61	13/7	NR	Tongue	(AJCC3) Stage I: 2 Stage II: 3 Stage III: 7 Stage 4: 8	47 months
Survival impact of nodal disease in hard palate and maxillary alveolus cancer	Lin and Bhattacharyya (2009)	2009	USA	R	725	70.9	46.1/53.9	1988–2004	Maxillary alveolus: 411 Hard palate: 314	(AJCC6) T1N0: 141 (Stage I); T1N1: 4 (Stage III) T2N0: 166 (Stage II), T2N1: 16 (Stage III), T3N0: 191 (Stage III), T3N1 (Stage III): 10, T3N2: 12 (Stage IVA), T4N0: 128 (Stage IV), T4N1:13 (Stage IVa), T4N2:25 (Stage IVa), T4N3:4 (Stage IVb)	T1–T4 96.5–49.3 months; N0–N3 73.7–5.5 months 5 years SR: T1: 60.3%, T2:43.3%, T3:46.6%, T4:26.4% N0: 46.2%, N1: 32.9%, N2:20.8%, N3:0%
Prognostic significance of primary tumor site in the oral cavity	Fries R., et al.	1980	Germany, Austria, Switzerland	R	585	64.5	450/135		Hard palate: 36 Alveolar process, maxilla: 83 Planum buccale: 20 Tonsil, pharyngeal wall pterygotemporal area: 23 Tongue: 85 Circumlingual sulcus: 84 Alveolar process, mandible: 121 Lower lip: 113 Upper lip and angle of mouth: 9 Buccomaxillary sulcus: 4 Bucco‐mandibular sulcus: 4 Soft palate: 3	(UICC3) T1N0: 132 T1NX: 80 T2N0: 142 T2NX: 121 T3N0: 55 T3NX: 55	12 years and 11 months
Non‐smoking non‐drinking elderly females: a clinically distinct subgroup of oral squamous cell carcinoma patients	K. Koo, et al.	2013	Australia	R	169	65.5; 80.1; ±6.2	57.4%/42.6%	2007–2010	Tongue: 72 Maxillary alveolus: 20 Retromolar area: 27 Mandibular alveolus: 7 Mouth Floor: 22 Buccal mucosa: 15 Hard palate: 6	(AJCC6) Stage I/II: 98 Stage III/IV: 71	28 months
A population‐based study of factors associated with early versus late stage oral cavity cancer diagnoses	Groome et al. (2011)	2011	Canada	R	2033	60.6 (Stage I) 60.8 (Stage II) 62.9 (Stage III) 62.8 (Stage IV)	60.6/39.4 (Stage I) 67/33 (Stage II) 68.5/31.5 (Stage III) 69.3/30.7 (Stage IV)	1991–2000	Tongue: 1221 Mouth floor: 812	(AJCC6) Stage I: 668 Stage II: 551 Stage III: 289 Stage IV: 525	NR
Oral tongue cancer in patients < 45 years old: institutional experience and comparison with older patients	Siegelmann‐Danieli N., et al.	1998	USA	R	87	56.4; ±17.9	M	1985–1996	Tongue	(AJCC3) Younger group: Stage I: 33%, Stage II: 20%, Stage III: 14, Stage IV: 33% Older group: Stage I: 44%, Stage II: 19%, Stage III: 12%, Stage IV: 25%	29 months (younger group), 21.4 months (older group)
Influence of smoking, age and stage at diagnosis on survival after larynx, hypopharynx and oral cavity cancers in Europe: the ARCAGE study	Abrahão R., et al.	2018	Europe: Czech Republic, Germany, Greece, Italy, Ireland, Norway, United Kingdom, Spain, Croatia	CS	1210 HNC (460 OC)	59.5; ±2.7	329/131 (OC)	2002–2011	Oral cavity: 460 Larynx: 604 Hypopharynx: 146	(AJCC6) OC cases Stage I: 86 (18.7%) Stage II: 93 (20.2%) Stage III: 47 (10.2%) Stage IV: 134 (29.1%) Unknown: 100 (21.8%)	4.6 years 5‐years SR: 55% for OC 65% larynx 35% hypopharinx
Prognostic performance of TNM8 staging rules in oral cavity squamous cell carcinoma	Jawad et al.	2020	Ireland	R	297	NR	199/98	2000–2016	Tongue: 117, mouth floor: 82, tongue & mouth floor: 4, alveolus: 37, retromolar trigone: 24, buccal mucosa: 19, lip: 12, hard palate: 2	T‐classification (AJCC7) T1 142, T2 82, T3 21, T4 52|N‐classification (AJCC7) Nx 82, N0 114, N1 42, N2a 0, N2b 44, N2c 15, N3 0|T‐classification (AJCC8) T1 72, T2 120, T3 41, T4 64|N‐classification (AJCC8) Nx 82, N0 114, N1 34, N2a 8, N2b 16, N2c 4, N3a 0, N3b 39	45 months
Validation of the American Joint Committee on cancer staging in squamous cell	Yung et al.	2021	Australia	R	297	66	202/95	2004–2019	Lip	(AJCC8) Stage I: 200, Stage II: 12, Stage III: 71, Stage IV: 14	NR
Comparative study on classifications of AJCC 8th and 7th in the patients with tongue squamous cell carcinoma	Yu et al.	2023	China	R	208	57.9; ±12.4	146/62	2019–2020	Tongue	(AJCC7) stage T1: 101; stage T2: 107 (AJCC8) stage T1: 69; stage T2: 104; stage T3: 35	NR
Squamous cell carcinoma of the mobile tongue in young adults: a Swedish Head & Neck Cancer Register (SweHNCR) population‐based analysis of prognosis in relation to age and stage	Jonasson et al.	2023	Sweden	R	1416	18–39 (36M, 46F), 40–64 (338M,238F), 65–74 (223M, 201F), > 75 (149M,195F)	746/680	2008–2017	Tongue	TNM 0.82; T1–2, N0 1017 (71.3%) 59 (72.0%) 410 (71.2%) 300 (70.8%) 248 (72.1%), T1–2, *N*+ 149 (10.4%) 7 (8.5%) 65 (11.3%) 41 (9.7%) 36 (10.5%); T3–T4, N0 97 (6.8%) 7 (8.5%) 29 (5.0%) 34 (8.0%) 27 (7.8%); T3‐T4, *N*+ 156 (10.9%) 9 (11.0%) 70 (12.2%) 46 (10.8%) 31 (9.0%); Missing 7 (0.5%) 0 (0.0%) 2 (0.3%) 3 (0.7%) 2 (0.6%); Stage 0.67; I 587 (41.2%) 34 (41.5%) 251 (43.6%) 168 (39.6%) 134 (39.0%); II 429 (30.1%) 25 (30.5%) 158 (27.4%) 132 (31.1%) 114 (33.1%); III 147 (10.3%) 11 (13.4%) 59 (10.2%) 41 (9.7%) 36 (10.5%); IV 253 (17.7%) 12 (14.6%) 105 (18.2%) 78 (18.4%) 58 (16.9%); Missing 10 (0.7%) 0 (0.0%) 3 (0.5%) 5 (1.2%) 2 (0.6%)	NR
Diagnostic delay of oral squamous cell carcinoma in two diagnosis centers in Cordoba Argentina	Morelatto et al.	2007	Argentina	R	70	59.9	46/24	1992–2004	Tongue: 27, gum: 8, toothless gum: 8, mouth floor: 10, buccal mucosa: 7, Retromolar trigone: 3, lip: 4, palate: 6	NR	NR
Malignancy grading of squamous cell carcinoma in the floor of the mouth related to clinical evaluation	Anneroth et al.	1986	Texas, USA	R	89	NR	56/33	1944–1978	Mouth floor	(AJCC1) Stage I: 39, Stage II: 38, Stage III: 12	5 years
Implication of the new AJCC pT classification of SCC of the lip comparing with other oral subsites	Toledo et al.	2021	Brazil	R	744	OSCC oral cavity 60.5 ± 11.5; OSCC lip 64.3 ± 13	OSCC oral cavity (474/175), OSCC lip (78/17)	2002–2017	Lips: 95, Retromolar trigone: 73, gum: 55, tongue: 255, buccal mucosa: 36, hard palate: 21, mouth floor: 209	(AJCC7) pT1 (*n* = 43, 45.3%): pT1: 18 (41.9%) pT2: 18 (41.9%) pT3: 7 (16.2%) pT4a: 0 (0.0%) pT2 (*n* = 34, 35.8%): pT1: 0 (0.0%) pT2: 17 (50.0%) pT3: 17 (50.0%) pT4a: 0 (0.0%) pT3 (*n* = 11, 11.6%): pT1: 0 (0.0%) pT2: 0 (0.0%) pT3: 11 (100.0%) pT4a: 0 (0.0%) pT4a (*n* = 7, 7.4%): pT1: 0 (0.0%) pT2: 0 (0.0%) pT3: 0 (0.0%) pT4a: 7 (100.0%) (AJCC8) pT1: 18 (18.9%) pT2: 35 (36.8%) pT3: 35 (36.8%) pT4a: 7 (7.4%)	Oral cavity 37, lip 33
Correlation between the magnetic resonance imaging features of squamous cell carcinoma of the buccal mucosa and pathologic depth of invasion	Baba et al.	2021	Japan	R	31	73.3 ± 8.3	26/5	2011–2020	Buccal mucosa	T1 6/31, T2 12/31, T3 9/31, T4a 4/31 (AJCC8)	NR
A retrospective analysis of squamous carcinoma of the buccal mucosa: an aggressive subsite within the oral. cavity	Lubek et al.	2013	USA	R	30	64	17/13	1992–2008	Buccal mucosa	pT1: 19, pT2: 7, pT3: 1, pT4: 3	50 months
Automatic upstaging of tongue squamous cell carcinoma with lateral extrinsic muscle involvment is not justified	Boland et al.	2013	United Kingdom	R	87	NR	42/45	1999–2008	Tongue	T1: 31, T2: 35, T3: 5, T4: 16, N0: 76, N1: 11, N2: 0, M0: 87 (AJCC7)	NR
Factors predicting the perineural invasion in carcinoma oral cavity	Dipti et al.	2023	India	R	553 PNI positive, 1163 PNI negative	48.76 PNI positive; 51.52 PNI negative	446/107	2009–2019	Buccal mucosa: 204, tongue: 161, lower alveobuccal: 101, lip: 4, mouth floor: 2	T1: 53, T2: 142, T3: 130, T4a: 228 (AJCC8)	NR
Oral tongue carcinoma: prognostic changes according to the updated 2020 version of the AJCC/UICC TNM staging system	Tagliabue et al.	2022	Italy	R	300	59	170/130	2010–2017	Tongue: 238 margin, 27 body, 23 ventral, 12 posterior third	Tis: 9; T1: 107; T2: 36; T3: 6 (AJCC7); t4a: 142; Tis: 9, T1: 84, T2: 44, T3: 104, T4a: 47 (AJCC8)	NR
Squamous cell carcinoma of the buccal mucosa: analysis of prognostic factors	Urist et al.	1987	USA	R	89	< 70: 36, > 70: 53	12/77	1959–1985	Buccal mucosa	T1: 22, T2: 32, T3: 20, T4: 14 (AJCC2)	NR
Clinicopathological factors related to cervical lymph node metastasis in a patient with carcinoma of the oral floor	Suzuki et al.	2007	Japan	R	48	58.4	45/3	1984–2006	Mouth floor	T1: 11, T2: 24, T3: 6, T4: 7 (UICC5)	5‐year SR Stage I: 71.4%; Stage II: 76.2%; Stage III: 50.3%; Stage IV: 40.3%.
Oral squamous cell carcinoma in the background of oral submucous fibrosis is a distinct clinicopathological entity with bettere prognosis	Gadbail et al.	2017	India	R	OSCC: 112; OSCC‐OSMF 105	OSCC: 53.39 ± 11.82; OSCC‐OSMF: 47.12 ± 12.07	OSCC: 77/35; OSCC‐OSMF: 82/23	2010–2013	(OSCC) buccal mucosa: 37, GB sulcus: 48, tongue: 5, retromolar trigone: 7, Palate: 6, lip: 8, mouth floor: 1; (OSCC‐OSMF) buccal mucosa: 48, GB sulcus: 20, tongue: 19, retromolar trigone: 9, palate: 4, lip: 3, mouth floor: 2	OSCC: Stage I: 2, Stage II: 18, Stage III: 32, Stage IV: 60; OSCC‐OSMF: Stage I: 14, Stage II: 34, Stage III: 23, Stage IV: 34 (AJCC6)	NR

Abbreviations: CS, cross‐sectional; NR, not reported; P, prospective; R, retrospective.

A total of 15,911 patients were included in the studies, diagnosed with oral cancer. Among these, the tongue was the most frequently involved site, with 5425 cases (34.1%), followed by the floor of the mouth with 1445 cases (9.1%) and the buccal mucosa with 1332 cases (8.4%). Tumors arising from the alveolus/gingiva/alveolar ridge accounted for 1203 cases (7.6%), while cancers of the lip and palate represented 703 cases (4.4%) and 610 cases (3.8%), respectively. Less frequently involved sites included the retromolar trigone/area with 279 cases (1.8%), the maxilla with 93 cases (0.6%), and the circumlingual sulcus with 84 cases (0.5%). Lesions involving the floor of the mouth in combination with the mandible accounted for 840 cases (5.3%). Additionally, a total of 121 cases (0.8%) were classified as other or not reported sites. It should be noted that, in addition to studies reporting unspecified or not reported subsites, one study did not provide a detailed subsite distribution and reported tumor location only as “oral cavity,” without further anatomical specification (Abrahão et al. 2020). Reports were single‐centre institutional series, with study populations drawn from North America (*n* = 9 studies), Europe (*n* = 6), Asia (*n* = 3), South America (*n* = 3) and Oceania (*n* = 2), while the remaining studies were multicentre or did not allow a clear regional attribution (*n* = 6).

### 3.3 Patient Demographics

Among studies reporting directly extractable mean age values, study‐level mean ages ranged from 33.8 to 73.3 years. Some studies, such as Jonasson et al. (2023) reported age‐group distributions rather than a single mean age and were therefore not included in this specific summary (Table 1). Sex distribution was consistently reported and showed a clear male predominance: 9153 males versus 4353 females, corresponding to 67.8% male overall. Summary of study characteristics, patient demographics, and clinicopathological data across included studies are reported in Table 2.

**TABLE 2:** Summary of study characteristics, patient demographics, and clinicopathological data across included studies.

Study	Diagnostic period	*N*	Mean age ± SD (years)	Gender (M/F) (%)	Main subsite(s)	Stage distribution	Staging system
Gamez et al. 2018	1980–2014	124	33.8 ± 5.4	59.7% (74/50)	Tongue (86.3%)	I: 65, II: 29, III: 9, IV: 12 Early (I–II): 75.6%	AJCC 7th
Jehn et al. 2019	NR	1486	60.0 ± 12.5	72.4%	Floor of mouth + mandible (56.5%), tongue (15%)	T1–T2: 893; T3–T4: 541 (TNM only)	UICC
Singhavi et al. 2020	2012–2014	863	50.91 ± 19.8	81.2% (701/162)	Tongue (42.3%), buccal mucosa (32.1%)	AJCC7: I: 216, II: 232, III: 134, IV: 281 Early (I–II): 51.9% AJCC8: I: 128, II: 212, III: 191, IV: 332 Early (I–II): 39.4%	AJCC 7th + 8th
Oji and Chukwuneke 2007	1998–2003	81	48.4 ± 16.1	59.3% (48/33)	Tongue (29.6%), floor of mouth (21%)	III: 70 (86%), IV: 11 (14%) Early (I–II): NR	NR
Alonso et al. 2018	1973–2012	1481	69.5 ± 13.1	50.5% (748/733)	Tongue (28.9%), alveolar ridge (21.4%), buccal mucosa (19.0%)	I: 208 (48.8%), II: 129 (30.3%), III: 50 (11.7%), IV: 39 (9.2%) Early (I–II): 79.1%	AJCC 7th
Abrahão et al. 2020	2011–2017	1463	59.8 ± 12.2	81.0% (1185/278)	Oral cavity: 599 (40.9%)	I: 174, II: 190, III: 287, IV: 812 Early (I–II) OC: ~39.1%^a^	AJCC 7th
Ribeiro et al. 2000	1990–1994	110	57.5 ± 8.2	84.5% (93/17)	Tongue (53.6%), floor of mouth (46.4%)	I: 14, II: 20, III: 30, IV: 46 Early (I–II): 30.9%	UICC 4th (2nd rev.)
Kuriakose et al. 2000	NR	20	61	65% (13/7)	Tongue (100%)	I: 2, II: 3, III: 7, IV: 8 Early (I–II): 25%	AJCC 3rd
Lin and Bhattacharyya (2009)	1988–2004	725	70.9	46.1%	Maxillary alveolus (56.7%), hard palate (43.3%)	Stage I (T1N0): 141 Stage II (T2N0): 166 Early (I–II): 42.3%	AJCC 6th
Fries et al. 1980	NR	585	64.5	76.9% (450/135)	Lower lip (19.3%), alveolar process mandible (20.7%), Tongue (14.5%)	T1N0: 132, T1NX: 80, T2N0: 142, T2NX: 121, T3N0: 55, T3NX: 55 (TNM only)	UICC 3rd
Koo et al. 2013	2007–2010	169	65.5/80.1 ± 6.2	57.4%	Tongue (42.6%), retromolar area (16%), floor of mouth (13%)	I/II: 98; III/IV: 71 Early (I–II): 58.0%	AJCC 6th
Groome et al. (2011)	1991–2000	2033	60.6–62.9 (by stage)	60.6%–69.3% (by stage)	Tongue (60.1%), floor of mouth (39.9%)	I: 668, II: 551, III: 289, IV: 525 Early (I–II): 60.0%	AJCC 6th
Siegelmann‐Danieli et al. 1998	1985–1996	87	56.4 ± 17.9	NR	Tongue (100%)	Young: I 33%, II 20%, III 14%, IV 33% Older: I 44%, II 19%, III 12%, IV 25%	AJCC 3rd
Abrahão et al. (2018)	2002–2011	460 (OC)	59.5 ± 2.7	71.5% (329/131, OC)	Oral cavity (100%)	I: 86 (18.7%), II: 93 (20.2%), III: 47 (10.2%), IV: 134 (29.1%), Unknown: 100 Early (I–II): 39.0%	AJCC 6th
Jawad et al. 2020	2000–2016	297	NR	67.0% (199/98)	Tongue (39.4%), floor of mouth (27.6%)	TNM only (AJCC 7th + 8th) T1–T2 (AJCC7): 224/297 (75.4%) T1–T2 (AJCC8): 192/297 (64.6%)	AJCC 7th + 8th
Yung et al. 2021	2004–2019	297	66	68.0% (202/95)	Lip (100%)	I: 200, II: 12, III: 71, IV: 14 Early (I–II): 71.4%	AJCC 8th
Yu et al. 2023	2019–2020	208	57.9 ± 12.4	70.2% (146/62)	Tongue (100%)	AJCC7: T1: 101, T2: 107 AJCC8: T1: 69, T2: 104, T3: 35 (T‐classification only)	AJCC 7th + 8th
Jonasson et al. 2023	2008–2017	1416	By age group (18–39/40–64/65–74/> 75)	52.7% (746/680)	Tongue, mobile (100%)	I: 587 (41.2%), II: 429 (30.1%), III: 147 (10.3%), IV: 253 (17.7%) Early (I–II): 71.3%	TNM 0.82
Morelatto et al. 2007	1992–2004	70	59.9	65.7% (46/24)	Tongue (38.6%), floor of mouth (14.3%)	NR	NR
Anneroth et al. 1986	1944–1978	89	NR	62.9% (56/33)	Floor of mouth (100%)	I: 39, II: 38, III: 12 Early (I–II): 86.5%	AJCC 1st
Toledo et al. 2021	2002–2017	744	OC: 60.5 ± 11.5 Lip: 64.3 ± 13.0	OC: 73.0% (474/175) lip: 82.1% (78/17)	Tongue (34.3%), Floor of mouth (28.1%), Lip (12.8%)	pT1–T2 (AJCC7): 81.1% pT1–T2 (AJCC8): 55.7%	AJCC 7th + 8th
Baba et al. 2021	2011–2020	31	73.3 ± 8.3	83.9% (26/5)	Buccal mucosa (100%)	T1: 6, T2: 12, T3: 9, T4a: 4 (T‐classification only)	AJCC 8th
Lubek et al. 2013	1992–2008	30	64	56.7% (17/13)	Buccal mucosa (100%)	pT1: 19, pT2: 7, pT3: 1, pT4: 3 (T‐classification only)	NR
Boland et al. 2013	1999–2008	87	NR	48.3% (42/45)	Tongue (100%)	T1: 31, T2: 35, T3: 5, T4: 16; N0: 76, N1: 11, N2: 0; M0: 87 (TNM only)	AJCC 7th
Dipti et al. 2023	2009–2019	1716	PNI+: 48.76 PNI−: 51.52	PNI+: 80.7% (446/107)	Buccal mucosa (38.9%), tongue (30.7%)	T1: 53, T2: 142, T3: 130, T4a: 228 (T‐classification only)	AJCC 8th
Tagliabue et al. 2022	2010–2017	300	59	56.7% (170/130)	Tongue (100%)	AJCC7: T1: 107, T2: 36, T3: 6, T4a: 142 AJCC8: T1: 84, T2: 44, T3: 104, T4a: 47 (T‐classification only)	AJCC 7th + 8th
Urist et al. 1987	1959–1985	89	< 70: 36 pts; > 70: 53 pts	13.5% (12/77)	Buccal mucosa (100%)	T1: 22, T2: 32, T3: 20, T4: 14 (T‐classification only)	AJCC 2nd
Suzuki et al. 2007	1984–2006	48	58.4	93.8% (45/3)	Floor of mouth (100%)	T1: 11, T2: 24, T3: 6, T4: 7 (T‐classification only)	UICC 5th
Gadbail et al. 2017	2010–2013	217 (OSCC: 112; OSCC‐OSMF: 105)	OSCC: 53.39 ± 11.82 OSCC‐OSMF: 47.12 ± 12.07	OSCC: 68.8% (77/35) OSCC‐OSMF: 78.1% (82/23)	Buccal mucosa + GB sulcus (OSCC: 75.9%; OSCC‐OSMF: 64.8%)	OSCC: I: 2, II: 18, III: 32, IV: 60 Early (I–II): 17.9% OSCC‐OSMF: I: 14, II: 34, III: 23, IV: 34 Early (I–II): 45.7%	AJCC 6th

*Note:* Early stage = Stage I–II combined where available.

Abbreviations: CS, cross‐sectional; GB, gingivobuccal; NR, not reported; OC, oral cavity; OSMF, oral submucous fibrosis; P, prospective; PNI, perineural invasion; R, retrospective.

^a^ OC‐specific subset only. Studies providing dual AJCC 7th/8th edition staging are indicated accordingly.

### 3.4 Age at Diagnosis

Of the 29 studies included in the qualitative synthesis, 20 contributed usable data to the descriptive summary of age at diagnosis presented in this section (Table 1). Twelve studies reported a single overall mean age (with SD inconsistently provided), whereas the remaining studies reported age using medians and/or categorical strata (e.g., age groups or restricted cohorts). Among studies reporting a single overall mean age (combined *n* = 4978), mean age at diagnosis ranged from 33.8 to 73.3 years, with a sample‐size‐weighted mean of 64.6 years across those datasets. This variability in mean age largely reflects differences in study design, as some investigations specifically focused on early‐onset OSCC with age‐restricted inclusion criteria. Mean ages in large, mixed‐age cohorts were generally in the sixth‐to‐seventh decade, while cohorts enriched for younger patients reported substantially lower mean values. Overall, age reporting was sufficiently consistent to describe ranges and central tendencies but not uniformly structured across all studies.

#### 3.4.1 Stage at Diagnosis

Staging data were also reported using non‐uniform formats and classification frameworks (e.g., full Stage I–IV distributions vs. grouped stages or TNM components, and different AJCC/UICC editions), limiting direct aggregation across 20 studies. Ten studies provided complete Stage I–IV counts, yielding an aggregate of 6950 staged OSCC cases. Within this subset, Stage I accounted for 2145/6950 (30.9%), Stage II for 1622/6950 (23.3%), Stage III for 1158/6950 (16.7%), and Stage IV for 2025/6950 (29.1%), corresponding to early‐stage (I–II) 3.767/6.950 (54.2%) and advanced‐stage (III–IV) 3183/6950 (45.8%) disease. Additional studies reported staging in alternative forms: one cohort provided grouped distributions (Stage I/II: 98 vs. Stage III/IV: 71), one series reported only advanced‐stage categories (Stage III: 70; Stage IV: 11), and another reported partial stage distributions (Stage I: 39; Stage II: 38; Stage III: 12, with Stage IV not specified). To formally assess the contribution of staging system heterogeneity to observed variability in stage distributions, five studies in this review classified the same patient cohort under both a pre‐8th edition AJCC/UICC framework and the AJCC 8th edition (Jawad et al. 2020; Yu et al. 2023; Toledo et al. 2021; Tagliabue et al. 2022; Singhavi et al. 2020) (Table S3). Across all five studies, application of AJCC 8th edition to the same dataset consistently produced a reduction in the proportion of early‐T cases relative to the earlier edition, with absolute differences in T1–T2 proportion ranging from −5.2% to −25.3%. The only study providing complete Stage I–IV distributions under both editions (Singhavi et al. 2020) showed a reduction in early stage disease from 51.9% under AJCC 7th edition to 39.4% under AJCC 8th edition (Δ = −12.5%). Where stage groups were not reported, TNM component reporting was available in at least one large cohort (total *n* = 1440 with TNM data), including T1–T2: 893, T3–T4: 541, N0: 832, N1: 611, M0: 1427, and M1: 13, with variable missingness across TNM fields (Table 1).

### 3.5 Assessment of the Risk of Bias and Publication Bias

The results regarding publication bias are presented in Figures 2 and 3. The follow‐up domains were rated as low risk of bias for all studies, as the outcomes of interest (age and stage at diagnosis) were assessed at baseline as cross‐sectional measures, rendering this domain non‐applicable in the traditional sense. Following the evaluation of the risk of bias for each study according to the Newcastle‐Ottawa Scale, only one study (Gamez et al. 2018) showed a medium risk of bias.

**FIGURE 2:**
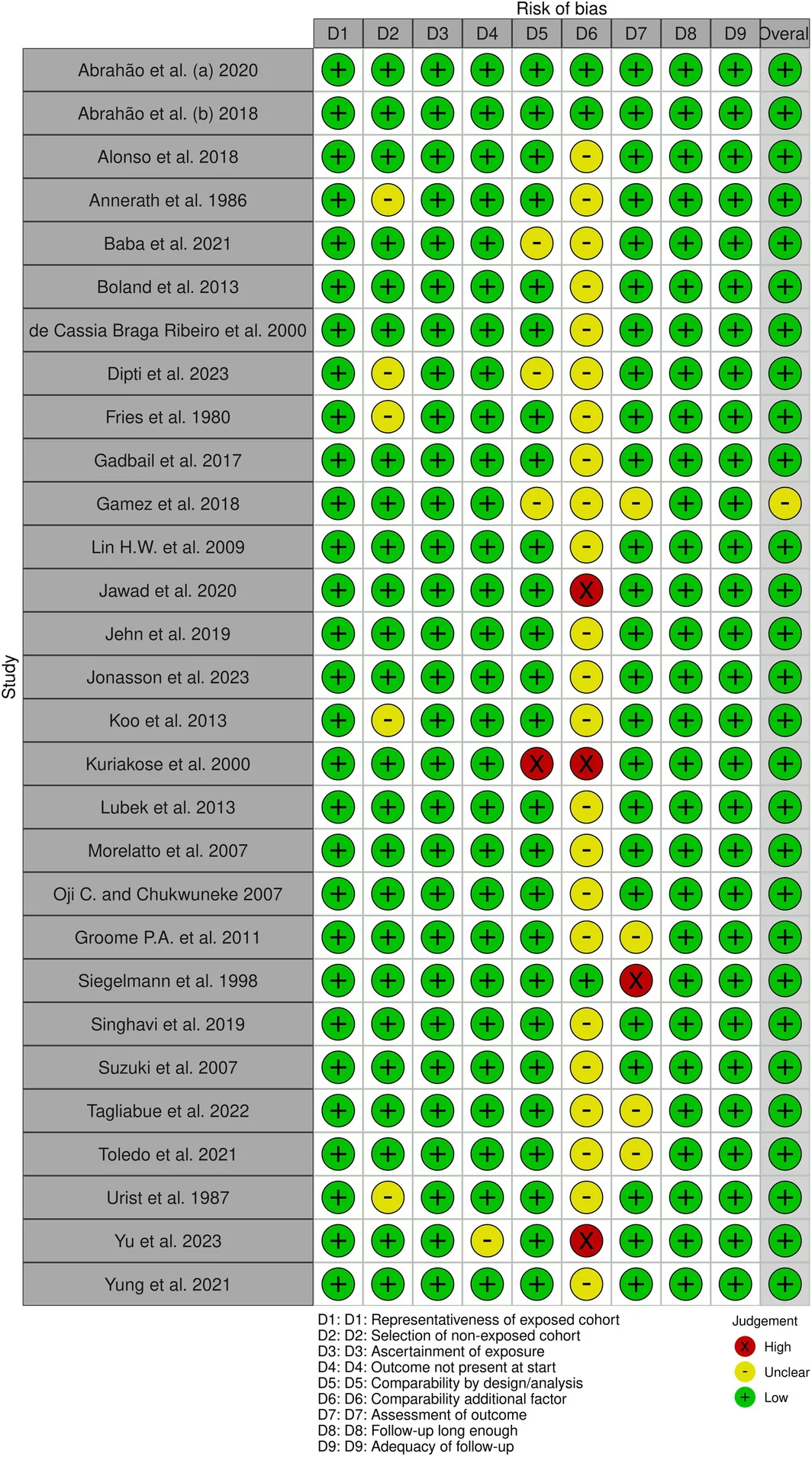
Risk of bias graph.

**FIGURE 3:**
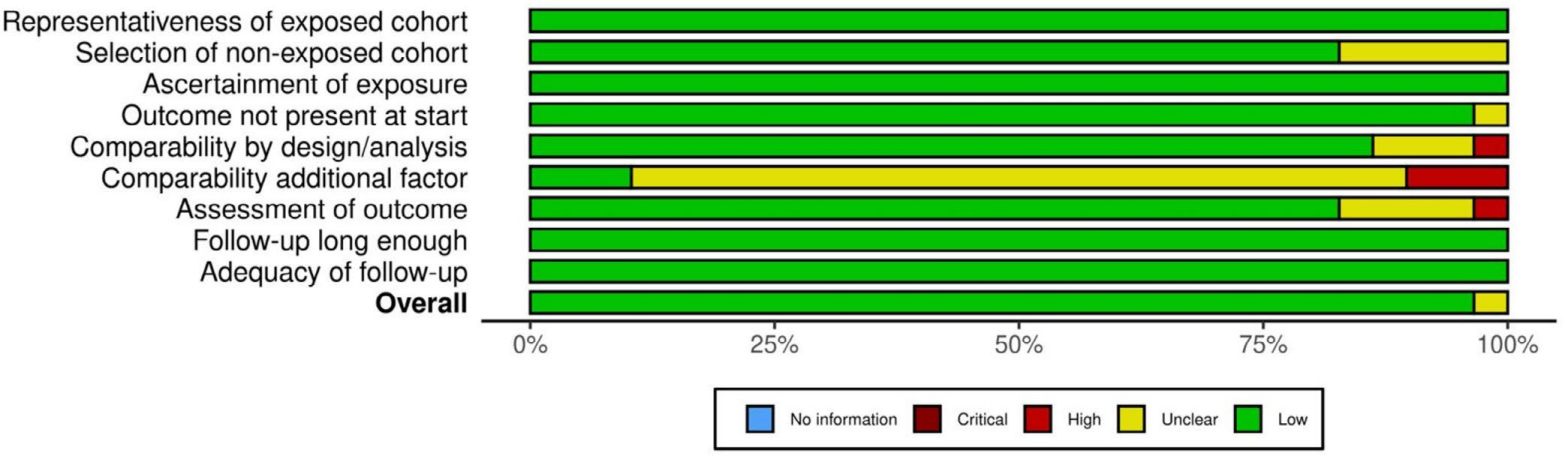
Risk of bias summary.

No major disagreements emerged during RoB assessment; inter‐rater reliability was substantial (Cohen's *κ* = 0.78). According to the Oxford Centre for Evidence‐Based Medicine (OCEBM) criteria, the overall level of evidence was rated as moderate (Level 2). This classification mainly reflects the study design that is typically feasible for this research question, which relies predominantly on retrospective cohort studies rather than randomized controlled trials or other experimental designs. Consequently, while the available evidence is informative and clinically relevant, its largely observational nature limits the possibility of achieving a higher level of evidence.

### 3.6 Results of the Meta‐Analysis

#### 3.6.1 Age at Diagnosis

The primary meta‐analysis (Figure 4), including all 11 studies eligible for quantitative synthesis (*n* = 3527), showed that the pooled mean age at diagnosis of oral squamous cell carcinoma was 57.42 years (95% CI: 54.51–60.33). A very high degree of between‐study heterogeneity was observed (*I* ^2^ = 99.2%, *p* < 0.01), indicating substantial variability across the included populations and study designs. Individual study estimates ranged from 50.34 (Gadbail et al. 2017) to 73.30 years (Baba et al. 2021), reflecting clinically meaningful differences in oral subsite distribution and cohort composition.

**FIGURE 4:**
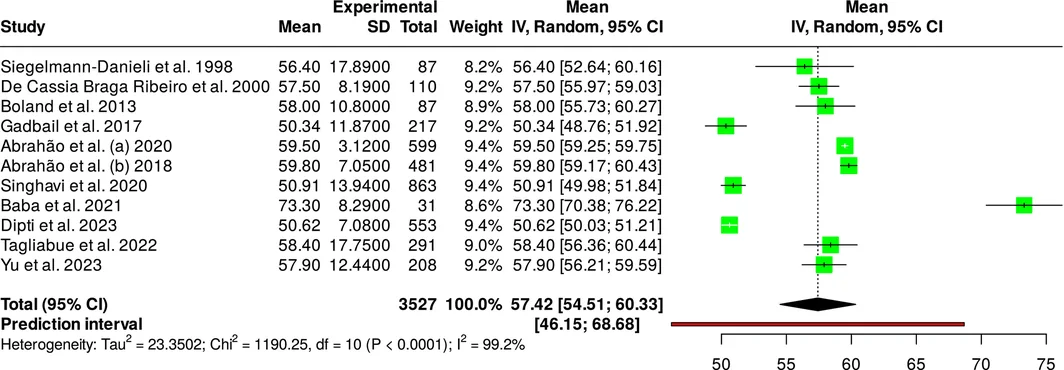
General meta‐analysis.

Visual inspection of the funnel plot (Figure 5) suggested a mild asymmetry, with smaller studies tending to report slightly lower mean ages at diagnosis. However, larger and more precise studies were symmetrically distributed around the pooled estimate. Given the high clinical and methodological heterogeneity among included studies, the observed asymmetry may reflect true between‐study variability rather than publication bias alone.

**FIGURE 5:**
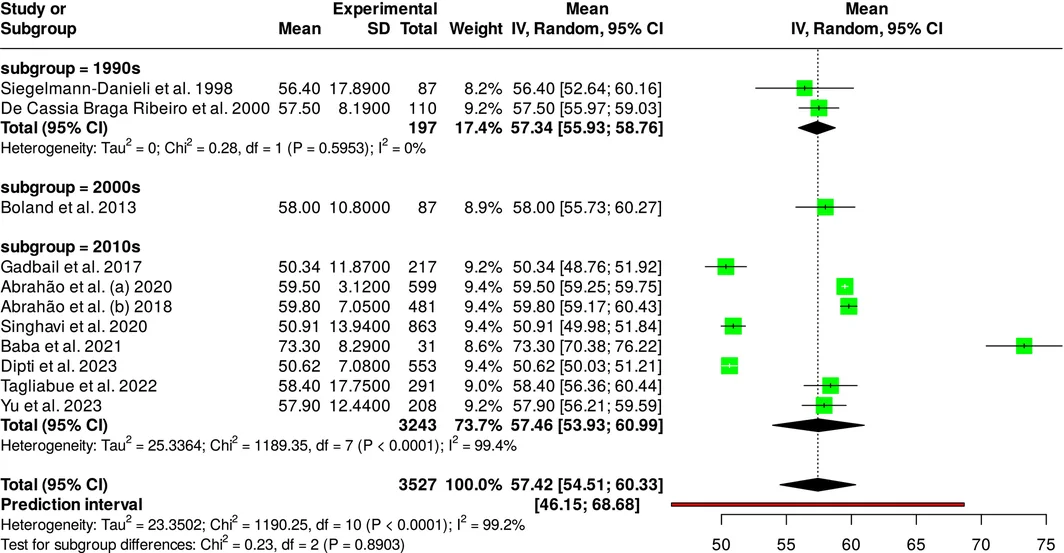
Subgroups meta‐analysis showing the mean age at diagnosis reported in the included studies. Studies are grouped by decade of publication (1990s, 2000s, and 2010s).

A subgroup meta‐analysis, stratified by decade of publication, was then performed to explore temporal trends (Figure 6). Studies conducted in earlier decades (1990s and early 2000s) reported higher mean ages at diagnosis, generally ranging between 57 and 58 years, with no heterogeneity in the earliest subgroup (*I* ^2^ = 0%, *p* = 0.60).

**FIGURE 6:**
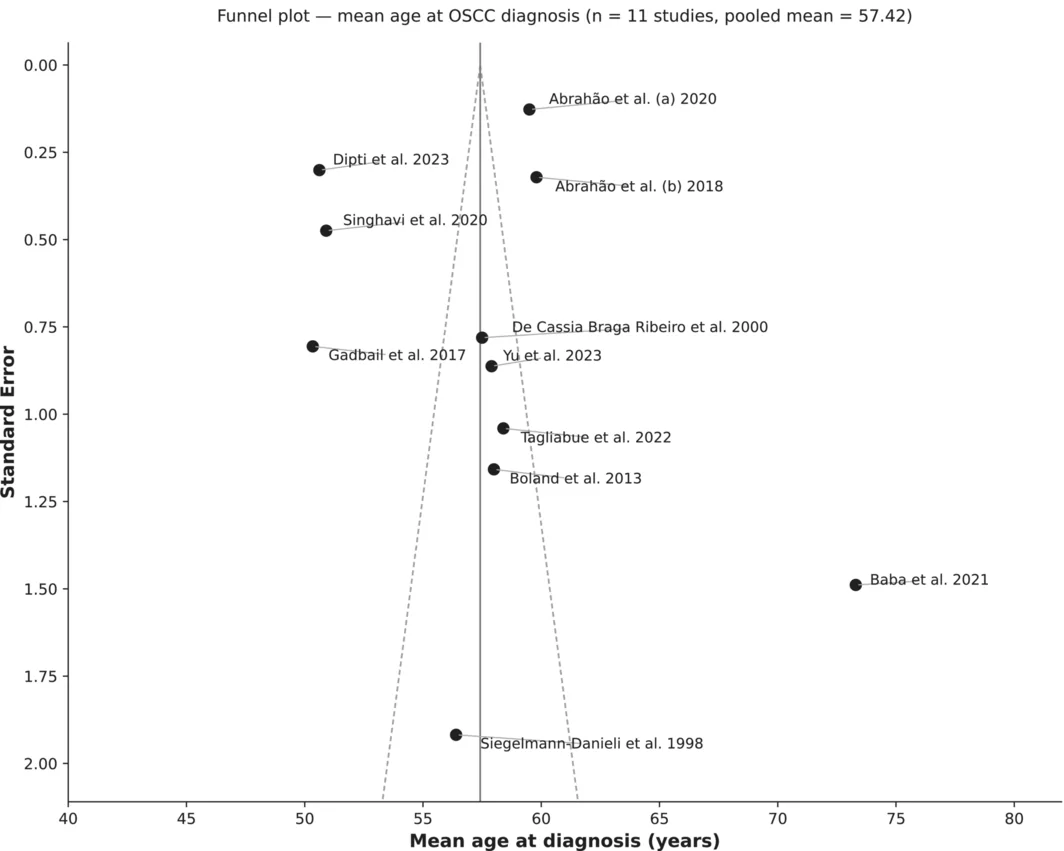
General funnel plot.

In contrast, studies published from the 2010s onward showed a lower pooled mean age at diagnosis, with several estimates converging around 50–52 years. The pooled estimate for the most recent decade was 54.64 years (95% CI: 50.49–58.80), although heterogeneity remained extremely high (*I* ^2^ = 99.1%, *p* < 0.01). Moreover, it can be observed a marked shift toward younger age at diagnosis, with the lowest pooled mean age decreasing substantially in more recent cohorts. Notably, for studies published in the 2010s, the pooled estimate was ~50.5 years (95% CI 50.49–58.80).

A sensitivity analysis restricted to studies with sample size ≥ 100 patients (8 studies, *n* = 3322; Figure 7) yielded a pooled mean age of 55.61 years (95% CI: 52.32–58.91; *I* ^2^ = 99.4%). This estimate was consistent with the primary analysis and directionally lower, suggesting that the pooled result is robust and not driven by small single‐centre series. Three studies were excluded from this sensitivity analysis due to sample size below the pre‐specified threshold (Baba et al. 2021; Boland et al. 2013; Siegelmann‐Danieli et al. 1998).

**FIGURE 7:**
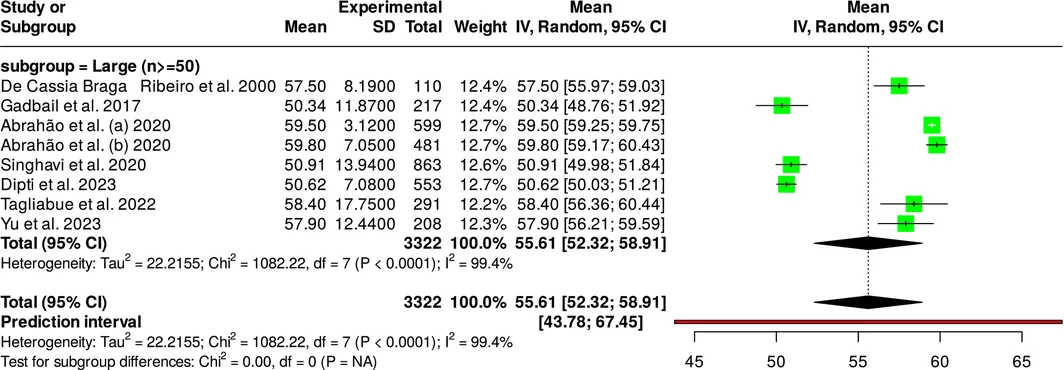
Sensitivity meta‐analysis restricted to studies with sample size ≥ 100 patients.

An additional subgroup meta‐analysis stratified by geographic region was then performed (Figures 8 and 9). Pooled mean age at diagnosis was 59.15 years (95% CI: 58.36–59.93; *I* ^2^ = 70.1%) in the Americas subgroup (4 studies, *n* = 1277), 58.22 years (95% CI: 56.70–59.74; *I* ^2^ = 0%) in the European subgroup (2 studies, *n* = 378), and 56.37 years (95% CI: 51.92–60.82; *I* ^2^ = 98.6%) in the Asian subgroup (5 studies, *n* = 1872). The test for subgroup differences was not statistically significant (*χ* ^2^ = 2.39, df = 2, *p* = 0.30).

**FIGURE 8:**
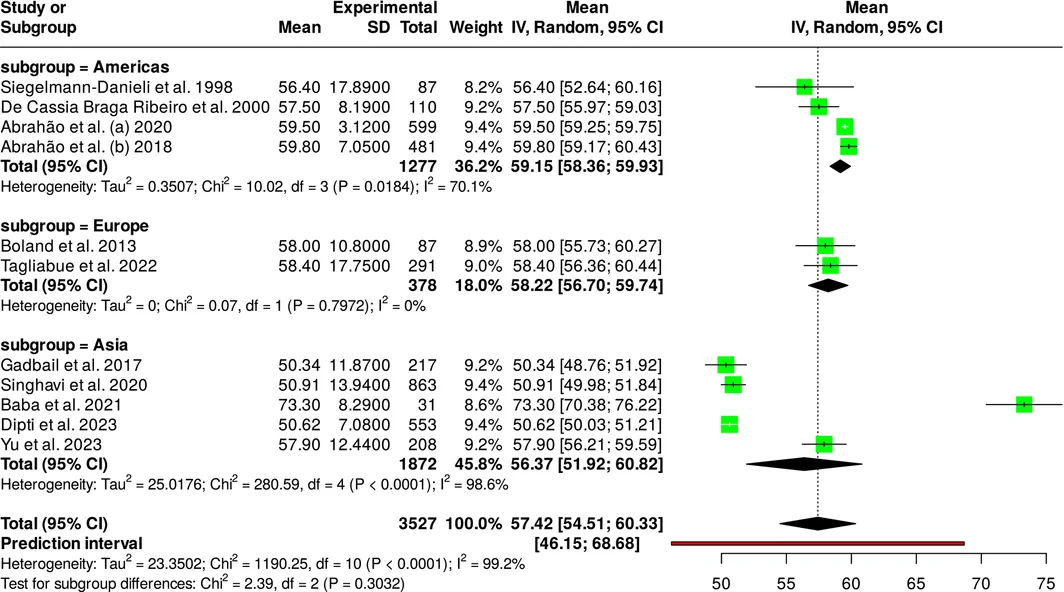
Subgroup meta‐analysis stratified by geographic regions (Americas, Europe, Asia).

**FIGURE 9:**
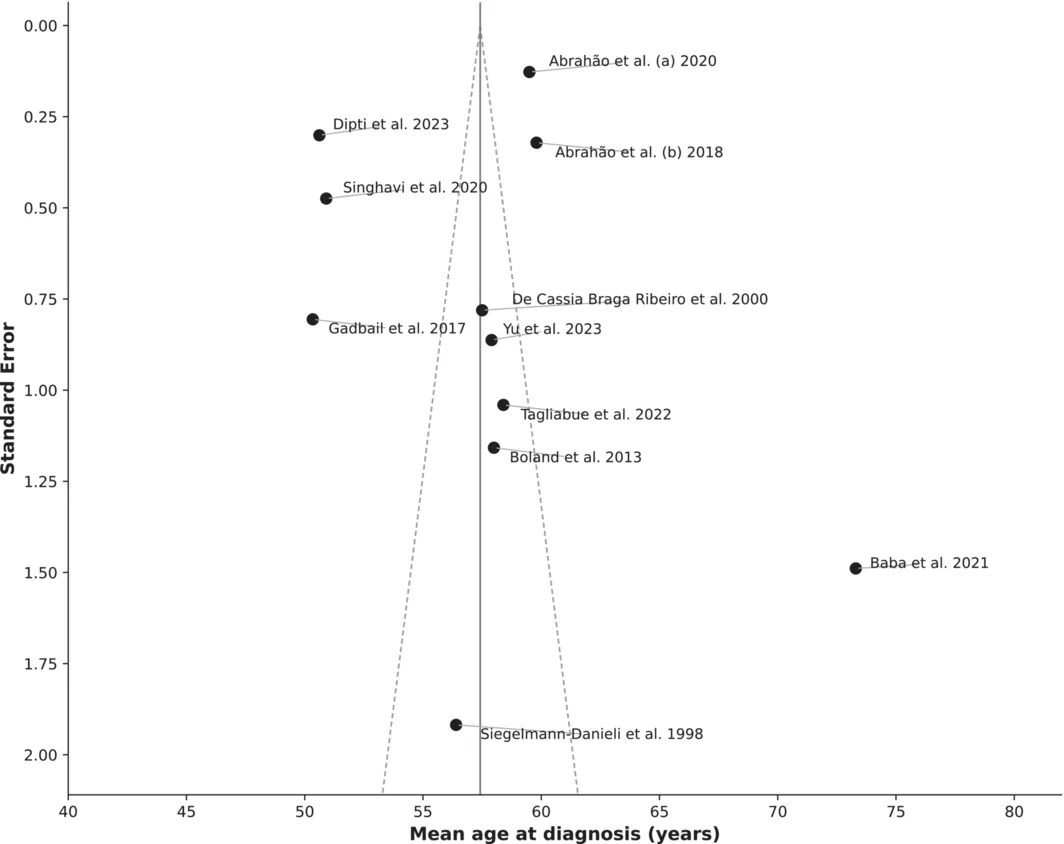
Funnel plot of the geographic subgroup meta‐analysis.

#### 3.6.2 Stage at Diagnosis

Meta‐analysis was not carried out because of the substantial heterogeneity among the included studies and the use of different staging and classification systems across study periods (e.g., changes in AJCC staging criteria). These methodological differences limited the comparability of the reported data and precluded a reliable quantitative synthesis. As a matter of fact, staging information were reported heterogeneously across the 29 included studies, using different formats (overall Stage I–IV distributions, grouped stages, or TNM categories) and different classification systems/editions (UICC/TNM and AJCC 3rd–8th editions). Ten studies provided complete Stage I–IV counts, yielding an aggregate of 6950 staged OSCC cases. Within this subset, Stage I accounted for 2145/6950 (30.9%), Stage II for 1622/6950 (23.3%), Stage III for 1158/6950 (16.7%), and Stage IV for 2025/6950 (29.1%), corresponding to early‐stage (I–II) 3.767/6.950 (54.2%) and advanced‐stage (III–IV) 3183/6950 (45.8%) disease. Several studies reported TNM distributions rather than stage groups; one large cohort reported T1–T2 893, T3–T4 541, N0 832, N1 611, M0 1427, and M1 13, with varying amounts of missing data. Some studies provided stage grouping only (e.g., I–II vs. III–IV) or reported predominantly advanced stages. Two studies reported stage distributions using both AJCC 7th and 8th editions on the same dataset, showing different stage allocations between editions. One study reported percentages for younger versus older groups, with Stage I–IV proportions of 33/20/14/33% in younger patients and 44/19/12/25% in older patients.

## 4 Discussion

### 4.1 Age Trend and Clinical Profile

This systematic review originates from a recurring clinical impression shared by many practitioners, namely: the age at diagnosis of oral squamous cell carcinoma appears to be decreasing. In daily clinical practice, an increasing number of cases seem to involve relatively younger patients, raising the question of whether this perception reflects a true epidemiological shift and the increase of new unknown risk factors. This systematic review investigated whether published evidence supports a clinically perceived increase in OSCC diagnoses among patients younger than commonly expected and often lacking traditional risk factors. OSCC has traditionally been considered a malignancy of older adults (Xu et al. 2019), consistent with the long latency required for cumulative carcinogenic exposure and the accumulation of genetic alterations; however, several contemporary series and multicentric datasets increasingly describe OSCC in young adults (Jonasson et al. 2023; Koo et al. 2013), often with a predominance of oral tongue involvement and with a substantial proportion of non‐smokers and non‐drinkers, raising the possibility of alternative or additional carcinogenic pathways in this subset. Although “young OSCC” is typically defined as < 45 years (with some variability across studies) (Dos Santos Costa et al. 2018; van Monsjou et al. 2013), the pooled evidence confirms that OSCC remains predominantly a midlife malignancy. The primary meta‐analysis of 11 studies (*n* = 3527) yielded a pooled mean diagnostic age of 57.42 years (95% CI: 54.51–60.33), consistent with a pre‐specified sensitivity analysis restricted to studies with *n* ≥ 100 (55.61 years, 95% CI: 52.32–58.91). Nevertheless, decade‐stratified analyses suggest a modest tendency toward younger age in more recent studies, with several point estimates clustering around 50–52 years. Qualitative evidence supports that early‐onset OSCC is clinically meaningful, particularly for oral tongue SCC, and that younger patients may show lower exposure to tobacco/alcohol while having broadly comparable survival once clinical factors are considered. Some studies included in our review, such as Gamez et al. (2018), Alonso et al. (2018), Anneroth et al. (1986), and Alonso et al. (2018), covered extended diagnostic periods (more than 30 years), which may have attenuated temporal changes in age distribution. To avoid potential bias in the meta‐analyses, these studies were not included.

### 4.2 Stage at Diagnosis and Reporting Heterogeneity

Staging data indicate that OSCC presents across a broad clinical spectrum and that advanced disease remains common. In the subset of studies reporting complete Stage I–IV distributions (*n* = 6950), early‐stage disease (I–II) accounted for 54.2% of cases, whereas advanced stages (III–IV) comprised 45.8%, with Stage IV alone representing ~29%, supporting the clinically relevant observation that late presentation remains frequent across contemporary cohorts and likely contributes to between‐study variability. However, interpretation of staging patterns over time was substantially constrained by non‐uniform reporting and definitions, including the use of different AJCC editions (3rd–8th), older UICC/TNM frameworks, and frequent reporting of grouped stages or partial TNM components. Decade‐based inspection suggested marked variability rather than a uniform temporal shift: in the 1990s, the UICC‐based series by Ribeiro et al. (2000) showed a predominance of advanced disease (Stage III–V 69.1%), whereas Siegelmann‐Danieli et al. (1998) reported broadly comparable stage distributions across age groups in oral tongue SCC (younger: Stage IV 33% vs. older: 25%); in the 2000s, Boland et al. (2013) provided TNM‐only data suggestive of earlier presentation (T1–T2 66/87; N0 76/87; M0 87/87), but Stage I–IV groupings could not be derived without additional assumptions; and in the 2010s, several cohorts reported substantial proportions of advanced disease (e.g., Gadbail et al. 2017; Abrahão et al. 2020), alongside more balanced distributions in others, reflecting heterogeneous case mix and settings. The analysis of five studies providing dual classification of the same cohort provides direct and quantitative evidence of the magnitute of stage migration associated with the introduction of AJCC 8th edition. The systematic reduction in early‐stage proportions across all five cohorts confirms that the DOI‐based (depth of invasion) refinements introduced in AJCC 8th edition produce consistent upstaging relative to prior frameworks, irrespective of the study population or subsite. The already cited AJCC 8th edition was introduced in 2017 and brought an important change to the T classification for oral cavity cancer by including depth of invasion alongside tumor size (Singhavi et al. 2020). This update was based on solid evidence showing that DOI is strongly associated with cervical lymph node metastasis and overall prognosis. This has a direct and important implication for the interpretation of our review: studies using AJCC 8th edition will sistematically report higher proportions of advanced disease compared with studies using earlier editions applied to comparable populations, even in the absence of any true change in clinical presentation. Critically, most studies did not report the stratified data required to determine whether the modest decrease in mean age observed in recent decades is accompanied by systematic changes in stage at diagnosis, either among younger patients or within tongue‐predominant cohorts, and the limited age‐stratified evidence does not consistently suggest earlier‐stage detection in young adults. Consequently, while advanced OSCC remains frequent and staging frameworks vary widely, the current evidence does not allow definitive conclusions on age–stage interactions or whether younger‐onset OSCC reflects earlier detection, changing risk profiles, or distinct biology; future studies should adopt uniform staging (preferably AJCC8), minimize missing TNM data, and provide age‐ and subsite‐stratified stage distributions to clarify whether temporal shifts in diagnostic age are coupled with changes in stage and metastatic burden.

### 4.3 Subsite Distribution and Interpretation

Subsite analysis confirmed a strong predominance of tongue involvement. Across 15,911 OSCC cases with available subsite information, the tongue was the most frequently involved site (34.1%), followed by the floor of mouth (9.1%) and buccal mucosa (8.4%). Oral tongue as the most affected subsite within the oral cavity, as previously underlined. Notably, this pattern appears even more pronounced in cohorts focusing on younger patients, where oral tongue SCC has been reported as the dominant presentation, as confirmed in the multicentre study by Gilligan et al. (2025) with 124 patients under 45 years old. From a clinical and biological perspective, attributing a younger diagnostic age primarily to improved detection is less compelling for OSCC, given its often aggressive behavior and potentially short window from subtle mucosal change to clinically overt disease (Hirsch et al. 2023). A more plausible interpretation is that the observed signal reflects a combination of population‐specific changes in risk profiles and etiologic heterogeneity (including non‐traditional exposures and host susceptibility), together with system‐level factors (awareness, access, and referral efficiency) that influence diagnostic timing. Baba et al. (2021) is included in the primary meta‐analysis alongside all other eligible studies. The marked variability in pooled estimates reflects genuine clinical and methodological heterogeneity. Notably, they reported a mean diagnostic age of 73.3 years, substantially higher than all other included studies. This finding is plausibly explained by the specific characteristics of that cohort and by the aim of the study: evaluation of RMI images in small cohort of Japanese patients (*n* = 31) exclusively composed of buccal mucosa SCC, a subsite with a distinct epidemiological and demographic profile compared to tongue‐predominant or mixed oral cavity cohorts. The advanced mean age in that series likely reflects population‐specific patterns in elderly Japanese patients rather than a generalizable OSCC trend. The influence of this study on the pooled estimate was formally assessed through the analysis restricted to studies with *n* ≥ 100, from which it was excluded on the basis of sample size. The consistency between the two meta‐analyses (57.42 years vs. 55.61 years) estimates supports the robustness and interpretability of the overall finding, while retaining all studies ensures completeness and transparency.

Although the observed decrease in mean age at diagnosis is modest, approximately 4 years, it should not be considered trivial from an epidemiological perspective. Given the very large absolute number of OSCC cases worldwide, even a small downward shift in the average age implies that a substantial number of carcinomas are being diagnosed at younger ages. In other words, a reduction of only a few years in the mean age requires a meaningful redistribution of cases toward earlier age groups. While this finding does not necessarily indicate a dramatic epidemiological transformation, it suggests that the presence of younger patients is not anecdotal and may represent a quantitatively relevant component of contemporary OSCC cohorts.

Overall, the available evidence does not support the existence of a clear and uniform global shift toward early‐onset OSCC. However, the findings are compatible with the possibility of a modest, population‐dependent tendency toward a younger age at diagnosis in certain contemporary cohorts, particularly those enriched for oral tongue SCC.

Considering the substantial heterogeneity observed, future investigations would benefit from more harmonized reporting standards, preferably adopting AJCC 8th edition staging, along with more complete TNM data and consistent age‐, subsite‐, and risk factor–stratified analyses. Prospective or large registry‐based studies with standardized methodologies may help clarify whether the observed patterns reflect true epidemiological changes or differences in case mix and reporting practices.

### 4.4 Quality Assessment

The quality assessment of the included studies revealed a moderate risk of bias overall, primarily related to the outcome domain of the Newcastle‐Ottawa Scale.

According to the Oxford Centre for Evidence‐Based Medicine classification, the overall level of evidence of this review was moderate (Level 2), as most included studies were retrospective cohort studies, with only one cross‐sectional and one prospective study contributing to the qualitative synthesis. This highlights the need for well‐designed prospective studies with standardized reporting of age‐specific incidence and staging data.

Overall, the funnel plot did not provide strong evidence of small‐study effects, but its ability to detect publication bias was limited by the number of studies and marked clinical/methodological heterogeneity.

### 4.5 Strengths and Limitations

Several limitations should be acknowledged, mainly related to substantial between‐study heterogeneity. The pooled estimate likely reflects an average across heterogeneous populations rather than a single, uniform epidemiological effect. Study‐level mean ages clustered into two main ranges (approximately 50–51 years and 58–60 years), suggesting systematic differences in case mix, such as geographic setting, prevalence of traditional risk factors, and oral subsite distribution, rather than random variation alone.

In addition, staging systems have changed over time, particularly across different AJCC/UICC editions. These modifications, including the introduction of DOI in AJCC 8th edition, may have led to stage migration and reduced comparability of stage distributions across decades.

It also remains unclear whether the modest decrease in mean age at diagnosis reflects a true epidemiological shift or, at least in part, increased awareness of oral cancer and earlier referral and diagnostic practices. Although some data suggest a relatively higher proportion of early‐stage diagnoses in recent cohorts, the available evidence does not allow us to determine whether improved sensitization alone accounts for the observed age trend.

Importantly, this review does not provide harmonized information on individual risk factors. Most included studies did not systematically report tobacco use, alcohol consumption, viral status, or other exposures in an age‐stratified manner, preventing firm conclusions regarding changes in risk profiles over time.

Most studies were not specifically designed to evaluate subsite‐specific OSCC, and anatomical location was often reported descriptively or in aggregated form without age or risk‐factor stratification, further limiting comparability.

Finally, although subgroup analyses stratified by TNM edition would ideally strengthen temporal comparisons of stage at diagnosis, the number of studies per edition in the present review was insufficient to conduct statistically meaningful subgroup analyses, with most editions represented by only two to three studies. This represents a further limitation of the available evidence base and underscores the need for future studies adopting uniform staging frameworks.

## 5 Conclusions

This systematic review synthesizes four decades of published evidence to evaluate whether OSCC is being diagnosed at a younger age and whether stage at presentation has changed over time. Overall, OSCC remains predominantly a midlife malignancy: the primary meta‐analysis of 11 studies (*n* = 3527) yielded a pooled mean diagnostic age of 57.42 years (95% CI: 54.51–60.33), consistent with a second analysis restricted to larger studies (*n* ≥ 100; 8 studies, *n* = 3322; pooled mean 55.61 years, 95% CI: 52.32–58.91). The robustness of this estimate across both analyses, combined with the improved funnel plot symmetry observed in the second analysis, strengthens confidence in the overall finding. Nevertheless, decade‐stratified analyses are compatible with a modest, population‐dependent trend toward younger age at diagnosis in certain contemporary cohorts, particularly those with tongue‐predominant or mixed oral cavity case mixes. The considerable between‐study heterogeneity observed in both analyses reflects genuine differences in geographic setting, subsite distribution, and risk factor profiles rather than methodological inconsistency alone.

Importantly, current staging data do not demonstrate a consistent parallel shift toward earlier‐stage presentation. This suggests that improved early detection alone is unlikely to fully explain the observed reduction in mean age at diagnosis, and that additional epidemiological or biological factors may be involved.

Future studies should prioritize large registry‐based or prospective designs with harmonized AJCC 8th edition staging, standardized reporting of oral subsites, and systematic age‐, sex‐, and risk factor‐stratified analyses. Such approaches will be essential to clarify whether emerging young‐onset OSCC reflects changing exposure patterns, distinct tumor biology, or a combination of both.

## Author Contributions

**Rita Antonelli:** conceptualization, investigation, writing – original draft, methodology. **Laura Ferrero:** investigation. **Cosimo Rupe:** software, formal analysis, validation, methodology. **Gioele Gioco:** writing – review and editing, visualization, validation, supervision. **Carlo Lajolo:** project administration, supervision, resources, conceptualization, investigation, writing – review and editing, visualization, validation, methodology. **Paolo Vescovi:** supervision, validation, visualization, writing – review and editing. **Francesco Scilla:** conceptualization, investigation, writing – original draft, data curation. **Romeo Patini:** supervision, software, formal analysis, writing – review and editing, validation, visualization, methodology. **Marco Meleti:** validation, visualization, writing – review and editing, supervision, project administration. **Giulio Tedeschi:** investigation. **Ilaria Giovannacci:** validation, writing – review and editing, visualization.

## Funding

The authors have nothing to report.

## Ethics Statement

The authors have nothing to report.

## Consent

The authors have nothing to report.

## Conflicts of Interest

The authors declare no conflicts of interest.

## Supporting information


**Table S1:** Search strategy.

**Table S2:** Excluded articles.

**Table S3:** Stage migration across AJCC/UICC editions in studies reporting dual classification.

**Data S1:** PRISMA 2020 checklist.

## Data Availability

The data that support the findings of this study are available from the corresponding authors (C.R. and G.G.), upon reasonable request.
