# Supplementary material for: Trends in Stage and Age at Diagnosis of Oral Squamous Cell Carcinoma Over Time: A Systematic Review and Meta‐Analysis
Source: Oral Dis. 2026 Jul 5;32(10):3004–28. doi: 10.1111/odi.70403 (PMC13768947; doi:10.1111/odi.70403)
Supplement: Supplementary file 1 [file ODI-32-3004-s001.docx]

| **Database** | **Search strategy** | **Records retrieved** |
| --- | --- | --- |
| PUBMED | ((((adult[MeSH Terms] OR aged[MeSH Terms] OR patient[MeSH Terms]) AND (mouth neoplasm[MeSH Terms] OR oral cancer[MeSH Terms] OR cancer, squamous cell[MeSH Terms] OR oral neoplasm[MeSH Terms])) AND (young adult[MeSH Terms] OR middle aged[MeSH Terms])) AND (cancer early diagnosis[MeSH Terms] OR cancer staging[MeSH Terms] OR neoplasm staging[MeSH Terms] OR early diagnosis[MeSH Terms] OR neoplasm grading[MeSH Terms])) AND (mouth[MeSH Terms]) | 1023 |
| Scopus | TITLE-ABS-KEY((adult OR aged OR patient* OR "young adult*" OR "young patient*" OR "middle aged" OR "under 50" OR "over 50") AND ("mouth neoplasm*" OR "oral cancer" OR "cancer, squamous cell" OR "oral squamous cell carcinoma" OR "oral neoplasm*" OR mouth) AND ("early diagnosis" OR "cancer staging" OR "neoplasm staging" OR "neoplasm grading" OR "stage at diagnosis" OR "age at diagnosis")) | 4053 |
| Web of Science | TS=((adult OR aged OR patient*) AND ("mouth neoplasm*" OR "oral cancer" OR "cancer, squamous cell" OR "oral neoplasm*") AND ("young adult*" OR "middle aged") AND ("cancer early diagnosis" OR "cancer staging" OR "neoplasm staging" OR "early diagnosis" OR "neoplasm grading") AND mouth) | 1207 |

**Supplementary Table S1. Search strategy**
