# Supplementary material for: Trends in Stage and Age at Diagnosis of Oral Squamous Cell Carcinoma Over Time: A Systematic Review and Meta‐Analysis
Source: Oral Dis. 2026 Jul 5;32(10):3004–28. doi: 10.1111/odi.70403 (PMC13768947; doi:10.1111/odi.70403)
Supplement: Supplementary file 2 [file ODI-32-3004-s002.docx]

**Supplementary Table S3. Stage migration across AJCC/UICC editions in studies reporting dual classification**

| Study | n | Metric | AJCC ≤7th Early (%) | AJCC 8th Early (%) | Δ (%) | Notes |
| --- | --- | --- | --- | --- | --- | --- |
| Singhavi et al. (2020) | 863 | Stage I-II | 51.9 | 39.4 | −12.5 | Stage I-IV fully reported for both editions |
| Jawad et al. (2020) | 297 | T1-T2 | 75.4 | 64.6 | −10.8 | T classification only; stage grouping not available |
| Toledo et al. (2021) | 95 | T1-T2 | 81.1 | 55.8 | −25.3 | Lip SCC subset; T classification only |
| Tagliabue et al. (2022) | 291 | T1-T2 | 49.1 | 44.0 | −5.2 | Tis excluded (n=9); T classification only |
| Yu et al. (2022) | 208 | T1-T3 | NR | NR | NR | 35 cases (16.8%) upstaged from T2 (AJCC7) to T3 (AJCC8); no T3/T4 in AJCC7 dataset |

*Abbreviations: AJCC, American Joint Committee on Cancer; SCC, squamous cell carcinoma; Tis, carcinoma in situ; T, tumour classification; Δ, absolute difference (AJCC 8th minus AJCC ≤7th, NR, not reported.*

*Notes: 'Early' refers to Stage I-II (for studies with complete stage grouping) or T1-T2 (for studies reporting T classification only). A negative Δ indicates a reduction in the proportion of early-stage/early-T cases under AJCC 8th edition, consistent with DOI-driven upstaging. Singhavi et al. is the only study in this review providing complete Stage I-IV distributions under both editions.*
